# Synovial Chondromatosis: A Narrative Review of Current Evidence on Diagnosis, Differential Diagnosis, and Management

**DOI:** 10.3390/medicina62071388

**Published:** 2026-07-18

**Authors:** Hassan Zmerly, Luigi Di Lorenzo, Federica Dellafiore, Alberto Righi, Laura Campanacci

**Affiliations:** 1 Department of Life Sciences, Health, and Health Professions, Link Campus University, 00165 Rome, Italy; l.dilorenzo@unilink.it (L.D.L.); f.dellafiore@unilink.it (F.D.); 2 Department of Orthopedics, Villa Erbosa Hospital, 40129 Bologna, Italy; 3 Unit of Surgical Pathology, Istituto Ortopedico Rizzoli, 40136 Bologna, Italy; alberto.righi@ior.it; 4 Third Orthopedic and Traumatologic Clinic Prevalently Oncologic, IRCCS Istituto Ortopedico Rizzoli, 40136 Bologna, Italy; laura.campanacci@ior.it

**Keywords:** synovial chondromatosis, synovial osteochondromatosis, loose bodies, synovial chondrosarcoma, arthroscopy, synovectomy, differential diagnosis, musculoskeletal tumors, magnetic resonance imaging, joint preservation

## Abstract

*Background and Objectives*: Synovial chondromatosis is a rare benign disease characterized by chondral metaplasia of the synovial membrane and the formation of intra-articular loose bodies. Although usually benign, delayed diagnosis may lead to progressive joint damage, recurrence, and, rarely, malignant transformation. *Materials and Methods*: A narrative review of the literature was conducted using the PubMed/MEDLINE database, focusing on studies published between January 2010 and December 2025. Eligible studies addressed the classification, epidemiology, clinical presentation, imaging findings, histopathology, differential diagnosis, treatment, recurrence, and prognosis of synovial chondromatosis. *Results*: Synovial chondromatosis most commonly affects large joints. Plain radiographs may show calcified loose bodies, whereas MRI is essential for detecting early non-calcified disease and assessing synovial involvement. Treatment depends on symptoms, disease extent, joint damage, and recurrence risk. Surgical removal of loose bodies with synovectomy remains the mainstay of treatment, while arthroplasty may be considered in advanced degenerative disease. *Conclusions*: This review provides an updated and clinically oriented synthesis of synovial chondromatosis, emphasizing early recognition, multidisciplinary diagnostic assessment, appropriate surgical management, and vigilant follow-up. Particular attention should be paid to recurrent disease and aggressive clinical or radiological features suggestive of malignant transformation.

## 1. Introduction

Synovial chondromatosis (SC), also called Reichel’s syndrome or synovial osteochondromatosis, is a rare benign disorder characterized by cartilaginous metaplasia of the synovial membrane resulting in the formation of multiple intra-articular cartilaginous nodules that may detach and become loose bodies within the joint cavity [1,2,3,4,5,6]. Once free, the cartilage fragments can continue to grow, nourished by synovial fluid, and potentially lead to joint destruction [7]. The loose bodies are composed of hyaline cartilage and may undergo endochondral ossification. This pathologic process usually remains benign, though very rarely it can involve malignant transformation [8].

SC most commonly affects large joints, particularly the knee and hip, although virtually any synovial joint, tendon sheath, or bursa may be involved. Because its clinical presentation is often nonspecific and imaging findings vary according to disease stage, diagnosis may be delayed for several years and the condition is frequently mistaken for more common degenerative, inflammatory, or neoplastic disorders [1,2,3,4]. Advances in magnetic resonance imaging (MRI), pathological characterization, and surgical techniques have considerably improved diagnostic accuracy and patient management over the last decade.

Despite the increasing number of case reports, case series, and observational studies, synovial chondromatosis remains a rare condition, and high-quality evidence is still limited. Furthermore, recent advances regarding imaging, histopathological diagnosis, differential diagnosis, recurrence, and surgical management have not been comprehensively integrated into an updated narrative review.

The aim of the present review is to provide an updated overview of the current evidence regarding the classification, epidemiology, pathophysiology, clinical presentation, diagnostic work-up, differential diagnosis, management, recurrence, and prognosis of synovial chondromatosis. Particular emphasis is placed on clinically relevant aspects that may facilitate early diagnosis, appropriate treatment selection, and recognition of features suggestive of malignant transformation.

## 2. Materials and Methods

A narrative review of the current literature on synovial chondromatosis was conducted using the MEDLINE/PubMed database. The literature search included articles published between 1 January 2010 and 31 December 2025. To ensure a comprehensive overview, landmark publications published before this period were also considered when they provided essential information regarding the classification, pathology, or historical evolution of the disease.

The following search terms were used, either alone or in combination: synovial chondromatosis, synovial osteochondromatosis, diagnosis, imaging, histopathology, management, treatment, recurrence, and malignant transformation.

The retrieved articles were screened according to their title and abstract. Full-text evaluation was subsequently performed for studies considered relevant to the objectives of this review. Additional publications were identified through manual screening of the reference lists of the selected articles to minimize the risk of missing relevant studies.

The inclusion criteria comprised English-language studies reporting original clinical data, systematic or narrative reviews, case series, and relevant case reports addressing one or more of the following topics: epidemiology, classification, clinical presentation, imaging findings, histopathology, differential diagnosis, treatment, recurrence, prognosis, or malignant transformation of synovial chondromatosis.

Articles published in languages other than English, conference abstracts without full text, duplicate publications, and studies not directly related to synovial chondromatosis were excluded.

Overall, 146 articles were initially identified through database searching. After title and abstract screening, 72 full-text articles were assessed for eligibility. Following application of the inclusion and exclusion criteria and additional manual reference screening, 50 publications were included in this narrative review.

Because of the heterogeneity of the available literature and the predominance of case reports and retrospective studies, a quantitative synthesis or meta-analysis was not considered appropriate. Therefore, the available evidence was analyzed descriptively, with particular emphasis on clinically relevant aspects regarding diagnosis, differential diagnosis, treatment strategies, recurrence, and prognosis.

## 3. Classification

Synovial chondromatosis (SC) is traditionally classified into primary and secondary forms [9]. Primary SC develops in an otherwise normal joint, in the absence of trauma or degenerative disease, and is currently considered a benign neoplastic process rather than a purely reactive condition. Cytogenetic studies have demonstrated recurrent clonal chromosomal abnormalities, particularly involving chromosome 6 [10], supporting its neoplastic origin. Secondary SC occurs in pre-existing joint diseases (e.g., osteoarthritis, post-traumatic, osteochondritis dissecans, or neuropathic arthropathy, etc.) due to chronic synovial irritation that causes formation of cartilaginous nodules [9].

Primary and secondary SC share the same mechanism of synovial cartilaginous nodule formation [11,12,13]. Synovial chondromatosis is generally considered a benign condition; however, rare cases of malignant transformation have been reported, typically in patients with a long-standing disease and multiple recurrences over several years [14,15,16,17,18].

## 4. Epidemiology

Synovial chondromatosis is a rare disease, with 1.8 cases per million per year [15]. SC typically affects adults between the third and fifth decades, with males affected approximately two to four times more often than females. SC most often involves large joints, with the knee being the most common location (approximately 60–70% of cases), followed by the hip, shoulder, elbow, ankle, and wrist [3,7]. Rare presentations have been documented in the temporomandibular joint and the spinal facet joint [1,14]. Tenosynovial chondromatosis is an uncommon extra-articular form that can affect bursae or tendon sheaths [16,19].

The principal risk factor for secondary SC is the presence of an underlying joint disorder. Mechanical stress has also been suggested to contribute to disease development, particularly in weight-bearing joints. In addition, several molecular pathways, including increased expression of bone morphogenetic proteins (BMPs), fibroblast growth factor-9 (FGF-9), vascular endothelial growth factor (VEGF), and inflammatory cytokines such as interleukin-6 (IL-6), have been implicated in the pathogenesis of the disease, although their precise role remains incompletely understood [20].

## 5. Clinical Presentation

Patients with synovial chondromatosis typically present with insidious monoarticular pain associated with progressive swelling, joint stiffness, and reduced range of motion [21,22]. Symptoms generally worsen over time and are often aggravated by joint movement or weight-bearing activities.

Mechanical symptoms, including locking, catching, clicking, or episodes of joint instability, are highly characteristic and are caused by intra-articular loose bodies. Crepitus may be detected during physical examination, while palpable nodules are occasionally present in superficial joints. Active synovitis may also result in local warmth, tenderness, and joint effusion [23].

Because these symptoms overlap with those of more common degenerative or inflammatory joint disorders, the diagnosis is frequently delayed. The interval between symptom onset and definitive diagnosis has been reported to average approximately five years [1].

Synovial chondromatosis is usually monoarticular. Multifocal involvement is uncommon and, when present, should raise suspicion for an underlying systemic disorder, such as rheumatoid arthritis. Progressive worsening of pain, rapid clinical deterioration, or repeated local recurrence should prompt further investigation to exclude malignant transformation into synovial chondrosarcoma.

## 6. Diagnostic Assessment

### 6.1. Imaging

**Figure 1:**
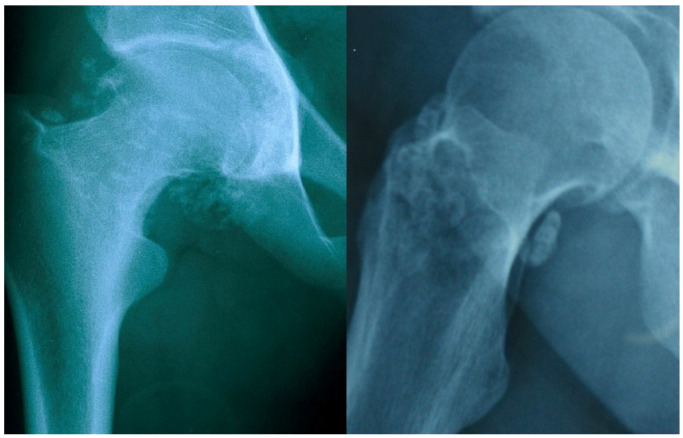
Imaging features of primary synovial chondromatosis of the hip. Radiographs demonstrate multiple calcified intra-articular osteochondral bodies associated with erosive changes and remodeling of the femoral head–neck junction.

Plain radiographs (X-rays) can show the characteristic multiple loose bodies, particularly after they have calcified or ossified (Figure 1). However, in the early stages of the disease, the cartilaginous nodules might not be calcified, with a normal image at X-rays [23]. Calcified nodules within the joint space will be visible on X-rays. These calcifications frequently exhibit a chondroid mineralization pattern that reflects the mineralization of cartilage tissue [24].

**Figure 2:**
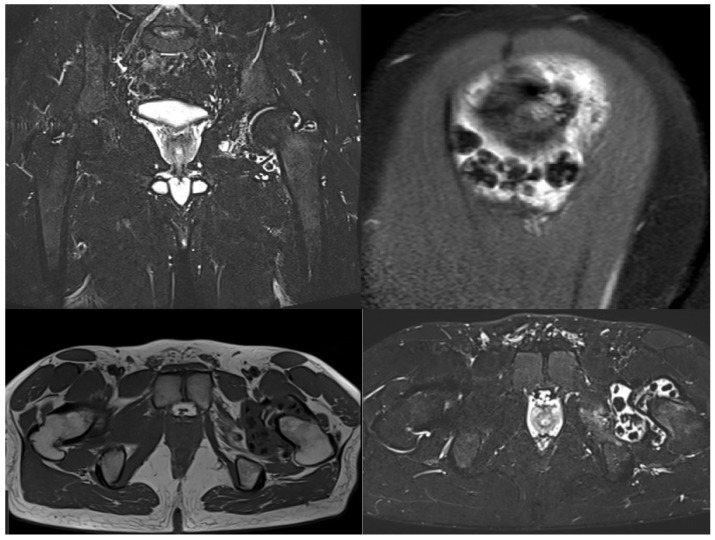
Coronal and axial MRI sequences show diffuse nodular synovial proliferation with numerous intra-articular loose bodies, partially calcified, exhibiting heterogeneous signal intensity and associated reactive changes.

Magnetic resonance imaging (MRI) can detect early or atypical cases even for uncalcified cartilage nodules that are not visible on X-rays [25]. SC typically presents as diffuse nodular synovial proliferation with intra-articular loose bodies (Figure 2) and is characterized by high T2 signal intensity within the joint and synovial tissue. After gadolinium contrast, MRI often shows synovial enhancement and thickening around the nodules, reflecting the active synovitis. MRI can also identify joint effusions and any extension of disease into nearby bursae or tendon sheaths. In later-stage disease, the MRI may reveal blooming artifacts or signal “drop-out” in nodules that have mineralized (due to calcification causing a low signal on all sequences) [26].

Computed tomography (CT) can help if there is doubt about calcification; CT is highly sensitive in detecting small calcifications and can confirm the presence of tiny ossified loose bodies that may not be visible on plain radiographs [1]. Ultrasound is less commonly used but can sometimes detect superficial loose bodies in accessible joints and guide aspiration if needed. Ultimately, the radiographic appearance of multiple intra-articular bodies strongly suggests synovial chondromatosis [27,28].

### 6.2. Histopathology

Histological examination is the gold standard for confirming the diagnosis of synovial chondromatosis, particularly in patients with atypical imaging findings or when malignant transformation is suspected [24,29,30].

**Figure 3:**
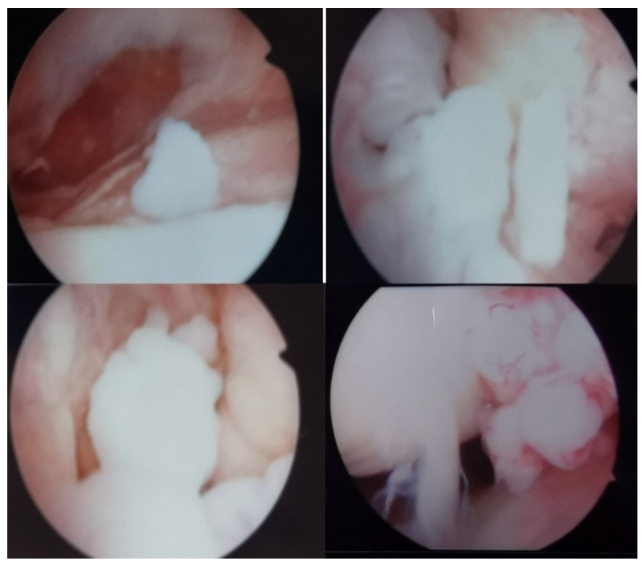
Arthroscopic images demonstrate multiple whitish osteochondral loose bodies, of variable size and morphology, within the joint associated with synovial hypertrophy and nodular proliferation.

Macroscopically, SC is characterized by multiple smooth, white cartilaginous nodules, that may remain attached to the synovium or become detached as free intra-articular loose bodies (Figure 3). Their size and degree of mineralization vary according to disease stage.

**Figure 4:**
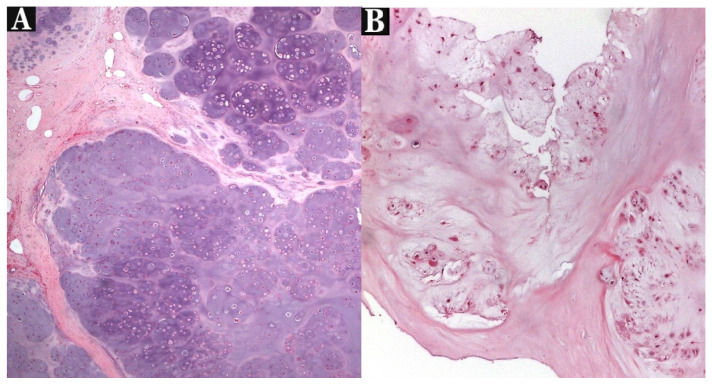
(**A**,**B**). Histological section of synovial chondromatosi. On haematoxylin and eosin at 50× of magnification, multiple cartilaginous nodules variably cellular with peripheral ossification are observed (**A**); at 200× of magnification clustered chondrocytes in a hyaline matrix without significant cytological atypia are evident (**B**).

Microscopically, the nodules consist of lobules of hyaline cartilage with increased cellularity and clustered chondrocytes embedded in a chondroid matrix; mild cytological atypia, binucleated chondrocytes, and focal calcification or endochondral ossification may be observed without implying malignancy [9,24,29] (Figure 4). Residual synovial lining tissue often occurs around the cartilage fragments.

The principal histopathological challenge is distinguishing benign SC from low-grade synovial chondrosarcoma, as both lesions may demonstrate increased cellularity and mild nuclear atypia. Features suggesting malignant transformation include infiltrative growth into adjacent bone or soft tissues, loss of the characteristic nodular architecture, diffuse cytological atypia, marked nuclear pleomorphism, increased mitotic activity, and permeative growth beyond the synovium [17,24,29,30].

Because isolated histological findings may occasionally overlap, definitive diagnosis should always integrate the clinical presentation, imaging findings, and pathological examination within a multidisciplinary setting. Rapid recurrence after apparently adequate treatment or progressive destructive imaging findings should prompt careful reassessment to exclude malignant transformation.

### 6.3. Differential Diagnosis

The differential diagnosis of synovial chondromatosis includes several benign and malignant disorders presenting with intra-articular loose bodies, synovial proliferation, or periarticular masses. Accurate diagnosis relies on the integration of clinical history, imaging, and histopathological findings (Table 1).

#### 6.3.1. Degenerative Joint Disease and Osteochondral Lesions

Loose bodies secondary to osteoarthritis, osteochondritis dissecans, or osteochondral fractures represent the most frequent differential diagnosis. In these conditions, loose bodies are usually fewer in number, irregular in morphology, and associated with advanced degenerative joint changes. In contrast, SC typically presents with numerous, relatively uniform cartilaginous nodules in joints that are initially well preserved [1,24].

#### 6.3.2. Inflammatory Arthropathies

Chronic inflammatory diseases, particularly rheumatoid arthritis, may produce synovial hypertrophy and fibrinous “rice bodies” that can mimic SC. Similarly, tuberculous arthritis may contain multiple rice bodies within the joint. Unlike SC, these lesions lack cartilaginous or calcified components and are usually associated with systemic inflammatory manifestations [21].

#### 6.3.3. Synovial Proliferative Disorders

Pigmented villonodular synovitis (PVNS), currently referred to as tenosynovial giant cell tumour (TGCT), typically presents with diffuse synovial proliferation but is characterized by hemosiderin deposition rather than cartilaginous nodules. MRI usually demonstrates low signal intensity on both T1- and T2-weighted sequences because of hemosiderin accumulation. Lipoma arborescens and synovial haemangioma should also be considered, although their characteristic fatty or vascular MRI appearance usually allows straightforward differentiation.

#### 6.3.4. Benign Cartilaginous Tumours

Soft tissue chondroma is an uncommon benign cartilaginous tumour arising outside the joint, most frequently within tendon sheaths. Unlike SC, it usually presents as a solitary extra-articular lesion without diffuse synovial involvement.

#### 6.3.5. Synovial Chondrosarcoma

The most important differential diagnosis is synovial chondrosarcoma, which may arise de novo or, rarely, through malignant transformation of longstanding recurrent SC. Clinical warning signs include rapidly progressive pain, repeated local recurrence, aggressive bone destruction, extra-articular soft tissue extension, and rapidly enlarging masses. Histopathological examination remains mandatory whenever malignant transformation is suspected [17,24,29] (Table 2).

## 7. Management

The management of synovial chondromatosis should be individualized according to the patient’s symptoms, the extent of synovial involvement, the presence of loose bodies, the degree of articular cartilage damage, and the likelihood of recurrence. Because of the rarity of the disease, current treatment recommendations are mainly based on case reports, retrospective case series, systematic reviews of observational studies, and expert opinion rather than high-level evidence [31,32,33,34,35,36,37,38,39,40,41,42,43,44,45,46,47,48,49]. This limitation should be considered when interpreting the available literature and highlights the need for higher-quality clinical studies.

In general, asymptomatic patients or those with minimal symptoms may initially be managed conservatively, whereas surgery remains the treatment of choice for symptomatic disease.

### 7.1. Conservative Management

Conservative treatment may be considered in carefully selected patients with mild symptoms, preserved joint function, and limited disease burden. This approach consists primarily of symptom control through nonsteroidal anti-inflammatory drugs (NSAIDs), analgesics, activity modification, and physiotherapy aimed at maintaining joint mobility and muscle strength [31,32].

However, conservative management does not eliminate intra-articular loose bodies or prevent disease progression and therefore has a limited role in primary synovial chondromatosis. Observation may be appropriate in asymptomatic patients or in individuals with substantial comorbidities who are poor surgical candidates.

In secondary SC, treatment should also address the underlying joint disorder, such as osteoarthritis or inflammatory arthropathy, which may contribute to ongoing synovial irritation.

At present, there is no pharmacological therapy proven to prevent cartilaginous nodule formation or disease recurrence. Experimental studies have suggested potential roles for molecular targets such as fibroblast growth factor-9 (FGF-9) or tumour necrosis factor-alpha (TNF-α), but these approaches remain investigational and should not be considered part of current standard clinical practice [20,30].

### 7.2. Surgical Management

Surgery remains the standard treatment for synovial chondromatosis and is indicated in patients presenting with persistent pain, mechanical symptoms, recurrent joint effusions, limitation of joint motion, or progressive joint damage [33,34,35,36,37,38,39,40]. The principal objectives of surgery are: removal of all intra-articular loose bodies; excision of pathological synovium; relief of mechanical symptoms; reduction in recurrence risk; and preservation of joint function. Whenever possible, all excised tissue should undergo histopathological examination to confirm the diagnosis and exclude malignant transformation [1].

Arthroscopic surgery is currently considered the preferred treatment for most cases involving large joints, particularly the knee. Compared with open surgery, arthroscopy offers lower surgical morbidity, less postoperative pain, faster rehabilitation, and excellent visualization of most joint compartments [35,39]. Current evidence suggests that arthroscopic loose-body removal combined with partial or complete synovectomy provides satisfactory functional outcomes with relatively low recurrence rates in appropriately selected patients.

Open synoviectomy remains indicated in patients with extensive synovial involvement, extra-articular extension, lesions located in anatomically difficult regions, or recurrent disease where complete arthroscopic excision is unlikely [40,41,42]. Although associated with longer rehabilitation and greater soft tissue dissection, open surgery may facilitate a more complete synovectomy, potentially reducing the risk of recurrence.

Both arthroscopic and open synovectomy are effective surgical options; arthroscopic surgery offers the advantages of smaller incisions, reduced postoperative pain, faster rehabilitation, and lower morbidity, making it the preferred approach for most intra-articular lesions of large joints, particularly the knee. However, complete synovectomy may be technically challenging in cases with extensive synovial involvement or disease affecting posterior compartments. Open synovectomy provides wider surgical exposure and may facilitate more complete removal of pathological synovium, particularly in extensive or recurrent disease and in anatomically complex locations. Nevertheless, it is associated with greater soft tissue dissection, longer recovery, and increased surgical morbidity [35,36,37,38,39,40].

Total joint arthroplasty should be reserved for patients with advanced secondary osteoarthritis, severe articular destruction, or irreparable cartilage damage. In these cases, arthroplasty simultaneously treats both degenerative joint disease and synovial pathology. Nevertheless, isolated cases of recurrent synovial chondromatosis after joint replacement have been reported, highlighting the importance of continued follow-up [41,42,43].

The current literature supports complete removal of loose bodies combined with synovectomy as the treatment associated with the lowest recurrence rates. However, the optimal extent of synovectomy remains controversial, and no randomized controlled trials directly comparing surgical techniques are currently available. Consequently, treatment decisions should be individualized according to disease location, extent of synovial involvement, surgeon experience, and patient characteristics [45,46,47,48,49].

The role of adjuvant therapies remains uncertain. Radiotherapy has been described only in isolated refractory cases and cannot currently be recommended as routine treatment because of the extremely limited available evidence [43,44].

Similarly, although several molecular pathways involved in synovial proliferation have been identified, targeted biological therapies remain experimental and require further investigation before clinical application.

## 8. Clinical Outcomes, Recurrence, and Prognosis

The clinical outcome of synovial chondromatosis largely depends on the extent of disease at diagnosis, the condition of the articular cartilage, and the completeness of surgical treatment. Most patients experience substantial pain relief and functional improvement following surgical removal of loose bodies combined with synovectomy [45,46,47,48,49].

### 8.1. Recurrence

Local recurrence remains one of the principal challenges in the management of synovial chondromatosis. Reported recurrence rates vary considerably among published studies, ranging from approximately 3% to 23%, reflecting differences in disease severity, anatomical location, duration of follow-up, and surgical technique [45,46,47].

Current evidence suggests that recurrence is more frequent after incomplete removal of pathological synovium or isolated loose-body extraction. Conversely, more extensive synovectomy appears to reduce recurrence rates, although high-quality comparative studies remain lacking [45,47,48,49,50].

Recent systematic reviews have reported recurrence rates of approximately 5.7% following arthroscopic synovectomy, compared with 31% after isolated arthroscopic loose-body removal, while open synovectomy showed intermediate recurrence rates of approximately 12%. Recurrence following total joint arthroplasty has also been described, although available evidence is limited [48,49].

### 8.2. Prognosis

The overall prognosis of benign synovial chondromatosis is generally favourable when early diagnosis and appropriate surgical management are achieved. Most patients recover satisfactory joint function and experience long-term symptom relief.

Nevertheless, delayed diagnosis may result in progressive cartilage degeneration and secondary osteoarthritis, potentially compromising functional outcomes and eventually requiring joint replacement.

The integrity of the articular cartilage at the time of surgery is considered one of the most important prognostic factors influencing long-term outcome.

### 8.3. Malignant Transformation

Although malignant transformation into synovial chondrosarcoma is exceptionally uncommon, clinicians should remain vigilant in patients presenting with longstanding disease, multiple recurrences, rapidly progressive symptoms, aggressive bone destruction, or extra-articular extension [17,29].

Because histological distinction between recurrent benign disease and low-grade chondrosarcoma may occasionally be challenging, multidisciplinary assessment combining clinical findings, imaging, and pathology is recommended whenever malignant transformation is suspected.

### 8.4. Follow-Up

Long-term clinical and radiological follow-up is recommended after surgical treatment because recurrence may occur several years after apparently successful management.

Follow-up should include regular clinical evaluation and imaging according to the affected joint and individual recurrence risk. Conventional radiographs are generally sufficient for routine surveillance, whereas MRI should be performed whenever recurrence or malignant transformation is suspected.

## 9. Conclusions

Synovial chondromatosis remains a rare but clinically important disorder that should be considered in the differential diagnosis of patients presenting with chronic monoarticular pain, swelling, and mechanical joint symptoms. Because early clinical manifestations are often nonspecific, delayed diagnosis is common and may lead to progressive cartilage damage, secondary osteoarthritis, recurrent disease, and, in exceptional cases, malignant transformation.

This narrative review provides an updated overview of the current evidence regarding the epidemiology, pathogenesis, diagnosis, differential diagnosis, histopathology, treatment, recurrence, and prognosis of synovial chondromatosis by integrating recent advances in imaging, pathology, and surgical management, it offers a practical framework to support clinical decision-making (Table 3).

Magnetic resonance imaging plays a pivotal role in the early detection of non-calcified lesions, while histopathological examination remains essential for confirming the diagnosis and excluding malignant transformation. Surgical removal of loose bodies combined with synovectomy continues to represent the cornerstone of treatment for symptomatic patients, although the optimal extent of synovectomy remains a matter of debate because of the limited availability of high-level evidence.

Future research should focus on prospective multicenter studies with standardized diagnostic criteria and long-term follow-up to better define recurrence patterns and optimize treatment strategies. Advances in high-resolution imaging, artificial intelligence-assisted image analysis, and molecular characterization of synovial lesions may improve diagnostic accuracy and facilitate earlier recognition of malignant transformation. Furthermore, collaborative international registries will be essential to generate higher-quality evidence for this rare disease and to establish evidence-based recommendations for patient management.

Finally, clinicians should maintain a high index of suspicion in patients with recurrent disease or rapidly progressive symptoms, as early recognition of malignant transformation is essential to ensure appropriate oncological treatment and improve patient outcomes.

## Figures and Tables

**Table 1:** Differential diagnosis of synovial chondromatosis: distinguishing clinical, imaging, and histopathological features.

Condition	Clinical Features	Imaging Findings	Histopathological Features
Primary synovial chondromatosis	Chronic monoarticular pain, swelling, stiffness, locking, mechanical symptoms	Multiple intra-articular cartilaginous or calcified loose bodies; synovial proliferation; MRI detects non-calcified nodules	Multiple hyaline cartilage nodules with clustered chondrocytes; variable calcification/ossification; no infiltrative growth
Osteoarthritis with loose bodies	Older patients, chronic degenerative symptoms	Joint space narrowing, osteophytes, subchondral sclerosis, few irregular loose bodies	Degenerative cartilage and osteochondral fragments without synovial cartilaginous metaplasia
Osteochondritis dissecans/Osteochondral fracture	History of trauma or young athletic patients; mechanical symptoms	One or few osteochondral fragments arising from the articular surface	Detached osteochondral fragment with viable or necrotic bone and cartilage
Rheumatoid arthritis/Tuberculous arthritis (rice bodies)	Polyarthritis or chronic inflammatory disease; possible systemic symptoms	Joint effusion with multiple small non-calcified rice bodies	Fibrinous rice bodies without hyaline cartilage
Tenosynovial giant cell tumour (TGCT, formerly PVNS)	Chronic swelling and pain; recurrent joint effusions	Diffuse synovial thickening with hemosiderin deposition; low signal (“blooming”) on MRI	Mononuclear cells, multinucleated giant cells, hemosiderin-laden macrophages
Soft tissue chondroma	Slowly growing extra-articular mass	Solitary calcified soft tissue lesion adjacent to tendon sheath	Benign hyaline cartilage without diffuse synovial involvement
Synovial chondrosarcoma	Progressive pain, rapidly enlarging mass, repeated recurrence	Aggressive bone destruction, extra-articular extension, invasive soft tissue mass	Infiltrative malignant cartilage with marked atypia, permeative growth, and soft tissue invasion

Abbreviations: MRI, magnetic resonance imaging; TGCT, tenosynovial giant cell tumour; PVNS, pigmented villonodular synovitis.

**Table 2:** Clinical, radiological and histopathological features suggestive of malignant transformation in synovial chondromatosis.

Feature	Benign Synovial Chondromatosis	Features Suggestive of Synovial Chondrosarcoma
Clinical course	Slowly progressive over months or years	Rapid clinical deterioration or sudden worsening after a long stable period
Pain	Mechanical pain, activity-related	Progressive, severe pain, often persistent or present at rest/night
Swelling	Slowly increasing joint swelling	Rapidly enlarging mass or marked soft tissue swelling
Recurrence	Occasional recurrence after incomplete synovectomy	Multiple or early recurrences despite apparently adequate surgical treatment
Joint function	Progressive stiffness and mechanical symptoms (locking, catching)	Rapid loss of joint function with increasing disability
Bone involvement	Pressure erosions or mild bone remodeling	Aggressive cortical destruction, medullary invasion, extensive bone erosion
Extra-articular extension	Rare	Soft tissue invasion or extra-articular extension
Radiographs/CT	Multiple calcified or ossified loose bodies; preserved bone architecture	Destructive bone lesions, ill-defined margins, cortical destruction, aggressive appearance
MRI	Multiple intra-articular nodules with synovial proliferation; well-defined lesions	Large heterogeneous infiltrative mass, bone marrow involvement, soft tissue extension, loss of normal tissue planes
Histopathology	Hyaline cartilage nodules with clustered chondrocytes, mild atypia, no infiltrative growth	Diffuse cytological atypia, loss of nodular architecture, permeative growth, bone and soft tissue invasion, increased mitotic activity
Recommended management	Synovectomy with loose-body removal and clinical follow-up	Biopsy and referral to a specialized musculoskeletal oncology center for multidisciplinary evaluation and oncologic treatment

Abbreviations: CT, computed tomography; MRI, magnetic resonance imaging.

**Table 3:** Overview of the main clinical features, diagnostic approach, treatment strategies, and follow-up of synovial chondromatosis.

Topic	Key Points
Epidemiology	Rare disease (1.8/million/year), knee most common site, male predominance
Clinical presentation	Pain, swelling, stiffness, locking, decreased ROM
Imaging	X-ray (calcified bodies), MRI (early disease), CT (calcifications)
Histopathology	Hyaline cartilage nodules, clustered chondrocytes, exclusion of malignancy
Differential diagnosis	OA, loose bodies, PVNS/TGCT, rice bodies, chondrosarcoma
Treatment	Conservative, arthroscopy, open synovectomy, arthroplasty
Recurrence	3–23%; lower after complete synovectomy
Follow-up	Clinical + imaging; biopsy if rapid recurrence

Abbreviations: ROM, range of motion; MRI, magnetic resonance imaging; CT, computed tomograghy; OA, osteoarthritis; PVNS, pigmented villonodular synovitis; TGCT, tenosynovial giant cell tumour.

## Data Availability

The original contributions presented in this study are included in the article. Further inquiries can be directed to the corresponding author.

## Abbreviations

The following abbreviations are used in this manuscript:

SC	Synovial chondromatosis
MRI	Magnetic resonance imaging
CT	Computed tomography
NSAID	Nonsteroidal anti-inflammatory drugs
